# Educating a Dentist to His Duty

**Published:** 1910-01-15

**Authors:** 


### EDUCATING A DENTIST TO HIS DUTY.

My Dear Harry: Ever since you first wrote to me asking suggestions about increasing the returns from your practice, I've been wondering whether I dare write you a very personal letter on the subject of educating patients. You furnished the material the last time I visited you, but I have feared to write, lest you take it as a personal criticism. I have decided, however, that you have sense enough not to do that, and that it may offer more definite suggestions than any other means makes possible.

One morning, as I loafed about your office, a well-dressed attractive lady came in and said that she wanted a tooth replaced on her upper partial plate. You sat her down in the chair and examined the articulation where the tooth had come loose, and then called me over to see. I looked at the tooth, of course, but I was so much struck with your own blindness as to your opportunities in that mouth that I had difficulty in restraining comment or in seeing the particular repair asked for. You took a little wax impression and bite of the area where the tooth had come away, and dismissed the patient. When she was gone I asked who she was. You replied, "That's Mrs. M—, wife of our leading grocer who has the fine store at the corner."

I didn't say anything for a while—I couldn't. I was so strongly moved to say unpleasant things about your business ability that I couldn't talk at all. I felt that you deserved to be poor and hardup and have your nose forever on the grindstone. And I knew that as long as you remained as blind to your opportunities as you were that morning nobody could help you.

Let me tell you what a rapid glance at that mouth revealed as Mrs. M's needs and your opportunities. She lost all her lower bicuspids and molars some years ago and some dentist had replaced them with a vulcanite lower partial plate that probably fit when made, but did not when I saw it. It moved about with every movement of the mouth and was held to place only when the upper teeth closed against it. As a result of lack of fit and adaptation, it was then pushing the tissues away from the distal sides of the cuspids, and the distal cementum and the places where the clasps rested on the labial surfaces were abraded and sensitive.

The natural lower anteriors were in place, but the gums were receding, and one or two were beginning to loosen. There were enough deposits on and between these teeth to account for the irritation at the gum margins and the gum withdrawal. Mrs. M— looked very healthy, with no visible excess tissue, and I don't believe her gum trouble was due to any systemic cause.

Of the upper anteriors, all were in place except the right lateral. The right central had a big gold contour filling which had evidently been in place a long time. A dark line to mesial of the filling, with a widening area of discoloration incisally, showed where decay was recurring, because the end of the tooth was worn away and had allowed leakage at the incisal edge. The upper right cuspid had a large amalgam filling on the distal surface, running around somewhat onto the buccal. The second molar was in place and looked sound save for an occlusal amalgam filling, apparently in good condition. The upper left cuspid had a full gold crown and the upper left third molar stood about where the second formerly stood. It had a very large amalgam filling, but seemed sound as a rock. All the upper teeth showed gum irritation and some deposits. The upper left lateral and the upper bicuspids and molars were on a vulcanite plate held to place with clasps that were wearing the enamel of the teeth they enclosed.

Evidently that mouth never presented itself to your vision as a whole. You saw it piecemeal, as its owner brought it to you for a repair of some particular spot that had given out. You probably never thought of putting that entire mouth into first-class condition. But I want to call your attention to the possibilities it offered. And these possibilities were not one-sided—provided you had good sense. They were of the greatest value to Mrs. M— in prolonged health and increased pleasure. They were valuable to you as a chance to sell your services, on a proper plane.

First, you should have put the soft tissues about all the remaining teeth in good shape. That means removing the deposits, polishing the teeth and massaging the gums. You knew about prophylaxis, because we'd been talking it over that very morning, but you evidently regarded it as a matter for discussion and not for practice, because when you had a chance like this you never mentioned it to the patient.

Next, you should have addressed yourself to the remedy of the havoc the partial plates were playing with that mouth. You saw their effects on the remaining tissues, both in forcing the soft tissues back and in abrading the remaining teeth. And that wear, as you might have seen, must require repair at no far distant date.

Furthermore, you might have seen at a glance by merely having the jaws closed, that neither she nor any other patient could properly masticate food on those plates. I'll venture that 90 per cent. of her hard foods were swallowed half chewed. Moreover, her jaws were coming closer together as the plates settled and her upper front teeth were already beginning to separate a little from the up-thrust of the lower anteriors, because the jaws were not properly separated in the back.

There are two or three systems by which a lower partial plate like this can be put in so firmly that the patient will hardly know them from her own. It requires crowning the lower cuspids in this case, and that is open to some esthetic objections, but the resulting advantages in this case certainly justify such a course. Mrs. M— is a lady in comfortable financial circumstances. She dresses well, she goes in good society, she has a good home. She can be shown the advantages of having this work done, and that in the best manner. This lower plate should by all means be on a gold base.

Coming to the upper jaw, you have chances for some work that will greatly benefit your patient. Suppose that you have already put the teeth and soft tissues in proper condition as to cleanliness. You will soon have to replace that big gold filling in the central, and it will then be so large as to disfigure her appearance. By taking it out and putting a Richmond crown on that tooth you can support from it the lateral which now is on the plate. That plate has no business whatever in that patient's mouth. Devitalize the upper right cuspid which now has the big amalgam filling, set a Richmond crown on it, crown the second molar and carry a bridge from the cuspid to the molar. At the same time bridge the missing lateral to the cuspid on the distal. Do the same on the left side, using that sound third molar as your posterior abutment. You will then have a mouth in good condition *as a whole.*

I once heard about a man who "couldn't see the forest for the trees." I guess that meant that he couldn't take any broad view of the situation because his mind was so taken up with the details. You suffer from that trouble, and since someone cured me of it, I find dentists everywhere who are still afflicted. They are so concerned with the details of an operation that it occupies their entire mind. That's the proper way to feel when you get to details, but it isn't proper at first.

I saw a railroad built once and I noticed that the engineers first looked over the ground *as a whole.* When they'd done that one gang of workers followed another, working down from the broad view of the engineers, to the narrow detailed view of the gang that drove spikes.

That's the way we should, as dentists, first go over the mouth as a whole, realizing the importance of its functions to the general health and vigor. Following the plans as a whole will naturally come plans in detail. And it will be time enough to concern ourselves with details when we come to their proper place. I guess patients aren't the only ones who need educational work. It looks to me as if we dentists need it quite as much as anyone else. We need to wake up.

I got a hint as to this broad viewpoint from a patient with a broader view than I had. He was a lawyer patient of mine for whom I had done some filling, etc. One day he came in, walked directly to the chair, sat down, and said: "I want you to give my mouth a careful, general survey. It isn't doing it's duty. I can't chew my food properly."

Well, sir, between the time he said that and the time when I looked at his mouth, I got a liberal education. I'd never really seen his mouth before, that is *as a mouth* with a series of intricate and important functions. I'd seen a tooth here and there and a cavity here and there, but I'd never seen the mouth. I hadn't seen "the forest for the trees."

You know an idea hits me hard when it does take hold, and I turned to the cabinet and fumbled something for a minute, while I let this idea of really looking at a mouth soak in. Why hadn't I thought of that before. What had I been doing all these years? Just tinkering—like a fellow who recently mended a tire for me and never saw that the wheel was out of true.

When I turned to the chair I was a better dentist than I'd ever been before. In fact I was a real dentist for the first time—a true stomatologist—who had the eyes and the ability to see that mouth as a whole and to study the repair of it as related to the health of the body.

It's immaterial what I did for that lawyer save that I repaired his mouth—not a tooth or teeth—but his mouth. And when I got through it functioned properly. What he did for me was immensely greater than what I did for him. He showed me by one swift, revealing flash what dentistry should be and how it differs from tinkering.

Get this viewpoint, Harry. View a mouth as a whole as the food gateway of the body, as a laboratory, where all food should be comminuted for digestion and the digestion of the starches should begin. Of course it's your duty to allay pain and make small repairs, but these are the details of the greater task.

Those two rows of teeth in proper condition and properly used mean health and vigor to the owner. They mean good digestion, proper assimilation, proper bowel elimination, and freedom from many of the ills that now beset us. More than physicians, more than neurologists, more than anybody else or all put together, the dentist holds the key to continued health by enabling the mouth to function properly, by teaching people to clean the mouth and keep it clean.

Come away from the trees and get a view of the forest. When you do, and join this perception of the needs of the mouth as a whole to good selling ability and your present skill, you won't have any trouble about getting money.

You'll learn to educate patients to your viewpoint and you'll teach them to pay good fees for the repairs you make. You'll rise to new levels, both professionally and financially, and can then do as some of my professional friends do now, lock your office for a couple of months each summer and enjoy those delights which are provided for those who have eyes to see and ears to hear.—Brother Bill in *Dental Digest.*

